# Transplant of cryopreserved mosquito gut microbiota reveals strain-specific patterns of microbial acquisition and assembly

**DOI:** 10.1093/ismehm/aazag010

**Published:** 2026-09-18

**Authors:** Holly L Nichols, Vishaal Dhokiya, Ananya F Hoque, Eva Heinz, Grant L Hughes, Kerri L Coon

**Affiliations:** Department of Bacteriology, University of Wisconsin-Madison, Madison, WI 53706, United States; Microbiology Doctoral Training Program, University of Wisconsin-Madison, Madison, WI 53706, United States; Departments of Vector Biology and Tropical Disease Biology, Centre for Neglected Tropical Diseases, Liverpool School of Tropical Medicine, Liverpool L3 5QA, United Kingdom; Departments of Vector Biology and Tropical Disease Biology, Centre for Neglected Tropical Diseases, Liverpool School of Tropical Medicine, Liverpool L3 5QA, United Kingdom; The Roslin Institute, Royal (Dick) School of Veterinary Studies, University of Edinburgh, Edinburgh EH25 9RG, United Kingdom; Strathclyde Institute of Pharmacy and Biomedical Sciences, University of Strathclyde, Glasgow G1 1XQ, United Kingdom; Departments of Clinical Sciences and Vector Biology, Liverpool School of Tropical Medicine, Liverpool, United Kingdom; Departments of Vector Biology and Tropical Disease Biology, Centre for Neglected Tropical Diseases, Liverpool School of Tropical Medicine, Liverpool L3 5QA, United Kingdom; Department of Bacteriology, University of Wisconsin-Madison, Madison, WI 53706, United States

**Keywords:** mosquito microbiome, microbiota transplant, *Aedes aegypti*, insect, host−microbe

## Abstract

Mosquitoes are important vectors of human pathogens, and their gut microbiome influences traits such as survival, fecundity, pathogen resistance, insecticide resistance, and vector competence. Manipulating this microbiome offers a promising strategy for reducing mosquito-borne disease. While 16S rRNA gene amplicon sequencing has revealed global patterns of gut microbial diversity, the factors driving these patterns remain poorly understood. Here, we used a community-transplantation approach that allows the collection, cryopreservation, and transfer of whole adult microbial communities into axenic (germ-free) mosquitoes to investigate the determinants of microbiome assembly and host–microbe interactions. Using reciprocal transplants between *Aedes aegypti* Liverpool strains maintained in isolation for nearly 50 years at the University of Wisconsin-Madison (UW) and the Liverpool School of Tropical Medicine (LSTM), we found striking strain-specific responses: UW mosquitoes exhibited altered bacterial composition and developmental defects, whereas LSTM mosquitoes did not. Transplant fidelity depended on donor community composition and recipient genotype, and we observed conserved assembly patterns across the mosquito life cycle. These results reveal how host genetics and microbial community structure interact to shape microbiome assembly and support the development of microbiome-based vector control strategies.

## Introduction

Vector-borne diseases account for a global disease burden of over 700 000 deaths annually [1]. One promising avenue to reduce pathogen transmission is manipulation of vector microbial communities [2–7]. Mosquitoes, the largest source of vector-borne diseases, contain microbiota that impact host development [8–10], survival [11–13], fecundity [14, 15], vector competence [16–19], and insecticide resistance [20–22]. Understanding host–microbe interactions could lead to new interventions, including bacteria that block pathogen replication in the mosquito, or bacteria with insecticidal properties.

The composition of the mosquito microbiome is shaped by diet [23–28], life stage [29–31], and diverse environmental factors [32–35]. For this reason, the mosquito microbiome is often considered transient. In contrast, much research on insect-associated microbes has focused on obligate, vertically transmitted symbionts [36–39] or stable communities reinforced by eusocial behaviors as seen in honey bees [40]. Given the diversity and abundance of holometabolous, non-eusocial insect species, transient associations with microbiota may be more common across the insect world [41–43]. These transient systems provide a unique opportunity to explore links between host genotype and microbial composition. However, it remains unclear whether coevolution occurs in systems where hosts interact with microbial partners for only part of their life cycle, and microbes may persist for many generations between encounters with a host.

Transience across generations or life stages does not preclude coevolution between hosts and microbes. Examples include nitrogen-fixing *Rhizobia* in legume nodules [44], bioluminescent *Vibrio fischeri* in the bobtail squid *Euprymna scolopes* [45], and nutritional symbionts of the genus *Caballeronia* in hemipteran insects [46, 47]. These associations all rely on environmental acquisition of microbial partners and impose fitness costs in their absence. However, such pairs employ strict partner-choice mechanisms such as secretion of mucus or antimicrobial peptides that exclude non-adapted microbes from colonizing specialized host organs.

Mosquitoes, like many dipterans, lack specialized organs for microbial colonization and occupy drastically different niches during immature and adult stages, reinforcing the transience of their microbial partners. Nevertheless, transient microbes provide clear benefits in insects including Diptera [48–50], Coleoptera [51], and Lepidoptera [52–54], often through digestion, nutrition, immune priming, or pathogen resistance. Whether these benefits arise from coevolution with specific microbes or from local adaptation to a consistent functional output of the microbial community (a “song” [55]), remains unclear. Short generation times and repeated exposure to similar habitats (detritus-rich stagnant water and floral nectar) enable frequent contact between mosquitoes and microbial partners. Common garden experiments have begun to reveal host effects on microbial composition: larvae of three *Aedes* species share a core microbiome when reared together [56], whereas co-reared *Aedes* and *Anopheles* larvae maintain distinct microbiota despite sharing many taxa [57]. These studies focus on the larval microbiota, which is critical for development but unlikely to persist into adulthood, when pathogen transmission occurs. Moreover, common garden experiments often rely on microbial input from the ambient lab environments, which can vary across cohorts [57]. Microbiota transplants offer greater control, enabling inoculation of different hosts with identical complex inocula across time [58].

Microbiota transplants have a long history in mammalian systems [59–61] but remain underutilized in insects. Gut microbiota transplants can reveal the role of microbial communities in processing diet-derived compounds that are otherwise inaccessible or toxic to the host, such as oxalate or tannins in wild rodents [62, 63] or diterpenes in pine weevils [64]. Transplants between distantly related organisms—or from environmental reservoirs into host-associated settings—can also test the strength of host filtering. For example, transplantation of donor pools from both mice and termites into germ-free cockroaches produced microbial communities resembling those of conventionally reared cockroaches [65].

In *Drosophila*, transplant experiments suggest that local host adaptation influences microbial assembly; flies receiving a mismatched microbiome exhibit developmental defects compared to both axenic individuals and those receiving a matched microbiome [66]. In mosquitoes, transplantation studies have shown that microbiota from larval habitats can be collected, cryopreserved, and reintroduced in mosquito-free laboratory microcosms [67]. LaReau *et al*. [68] inoculated axenic larvae with freshly field-collected water and observed increased bacterial diversity and larger temporal shifts during blood meal digestion compared to conventionally reared individuals. Similarly, transplantation of adult microbial communities from *Culex quinquefasciatus* or *Aedes aegypti* donors into recipient *Ae. aegypti* larvae produced microbial diversity resembling that of donor adults [69]. Most published methods describe transplantation within a single laboratory, as they require freshly generated donor material. Here, we introduce a method to cryopreserve adult mosquito-derived donor pools, enabling transplantation across laboratories and over time.

Laboratory husbandry of mosquitoes undoubtedly acts as a selective pressure on both hosts and their associated microbes. In this study, we used two colonies of *Ae. aegypti* Liverpool (LVP) strain mosquitoes, each reared in isolation for 50 years, to investigate how host−microbe dynamics shift over extended time scales. Previous work shows that microbial composition in newly established (<10 generations) *Ae. aegypti* lab colonies collected worldwide converges toward a common microbiome [70], underscoring the strong selective influence of laboratory environments on microbial communities. Moreover, conditions between insectaries can differ substantially: when four different *Ae. aegypti* lines were reared at two insectaries, insectary identity was a much stronger predictor of bacterial community composition than host genotype [71, 72]. These findings suggest that prolonged laboratory culture may lead to drift and eventual divergence of both host and microbial communities. Here, we introduce a method to cryopreserve, store, and share adult mosquito microbiota for transplantation into axenic larvae within a closed system. This approach allows us to: (i) assess transplant fidelity after extended storage at −80°C and (ii) examine the effects of rearing mosquitoes on a mismatched microbial community, probing the specificity of host–microbe interactions in two long-isolated Liverpool strain colonies.

## Materials and methods

### Mosquito provenance and rearing conditions

Mosquitoes reared at the Liverpool School of Tropical Medicine (LSTM) belong to the “Liverpool line” (LVP), originate from collections in West Africa in 1936, and have since been continually maintained at LSTM [73]. Insects from the LSTM LVP colony were shared with laboratories in the USA in the 1960s [74]. The current University of Wisconsin-Madison (UW) colony was brought from Liverpool to Iowa State University in 1977, was moved to Murray State University in 1978, and finally arrived at the UW in 1982 where it has since been continually maintained (B. Christensen, personal communication).

At LSTM, eggs from the Mosquito Symbiosis and Infection group LVP colony were hatched and reared in an insectary maintained at 27°C and 70% relative humidity, with a 12:12 h light:dark photoperiod including 1 h crepuscular periods. Approximately 20 pans of larvae were reared at a density of 150 larvae per pan, in 1 L of tap water. Larvae were fed ground tropical fish flakes (Tetra, Melle, Germany) *ad libitum*. Pupae were collected and allowed to emerge in non-sterile hard-shell cages. After emergence, adults were provided with 10% (wt/vol) sucrose solution *ad libitum*. When the cohort reached 3–4 days post emergence, females were collected from the cage following cold anesthesia for 10 min at 4°C.

At UW, the LVP strain of *Ae. aegypti* was conventionally reared under the following environmental conditions: constant temperature of 26.5 ± 1°C, 75 ± 10% relative humidity, and a 16:8 h light: dark photoperiod with 90 min crepuscular periods. Larvae were hatched in deoxygenated water. Females received defibrinated sheep blood (Hemostat Laboratories, Dixon, CA, USA) on Day 4 post-emergence. To begin the present experiment, eggs from the insectary colony were hatched and reared in an incubator (Percival Scientific, Perry, lA, USA) maintained at 28°C with a 16:8 h light:dark photoperiod. Twenty replicate pans of first instars (L1) were reared at a density of 300 larvae per aluminum pan. Larvae were kept in distilled water and fed ground tropical fish flakes (Tetra, Melle, Germany) *ad libitum*. Pupae were collected and allowed to emerge in non-sterile mesh cages. After emergence, adults were provided with 10% (wt/vol) sterile sucrose solution *ad libitum*. When the cohort reached 3–4 days post emergence, females were collected from the cage following cold anesthesia for 10 min at 4°C.

### Preparation of donor pools for cryopreservation

Donor pools were prepared in parallel on the same day at the UW and the LSTM. Prior to homogenization, donor mosquitoes were surface-sterilized in bulk within a 50 ml conical tube by gentle inversion in 70% ethanol for 5 min, followed by three 3-min rinses in sterile water. Each donor pool was generated by homogenizing 720 whole, sugar-fed adult female mosquitoes (3–4 days old). At UW, homogenization was performed for 20 s in a bead-beater with three 5-mm stainless steel beads; at LSTM, homogenization was performed for 1 min at 26 Hz using a TissueLyser. Mosquitoes were processed in groups of 18 in sterile screw-top tubes containing three stainless steel beads and 1 ml of PBS. Homogenates were centrifuged at 1000 rcf for 3 min to pellet debris, and 800 μl of supernatant from each homogenate was pooled to obtain 32 ml of combined homogenate. The remaining supernatant was retained for microbial composition analysis. The pooled homogenate (“donor pool”) was passed through a 5-μm polyethylene terephthalate mesh filter on ice. Of this filtrate, 2.4 ml was used immediately for the “unfrozen” treatment (see "Microbiota transplantation with unfrozen and cryopreserved donor homogenates"). The remaining filtrate was centrifuged at 3220 rcf for 20 min, after which the supernatant was discarded and the pellet gently resuspended in 2.96 ml of sterile 20% glycerol. Donor pools were aliquoted into twelve cryovials (240 μl each) and frozen at −80°C for future use. Six vials of frozen donor pool were shipped to the opposite laboratory the following day. Shipping times were 2 days from UW to LSTM and 15 days from LSTM to UW, with samples maintained on dry ice throughout transit.

### Microbiota transplantation with unfrozen and cryopreserved donor homogenates

To prepare larval water for the “unfrozen” donor pool treatment, 2.4 ml of filtered, unfrozen homogenate was immediately combined with 119.6 ml of sterile water. Four T25 vented culture flasks were each filled with 30 ml of this mixture and stocked with 30 newly hatched axenic first instars as described later in this section. Two control flasks were included: one containing sterile water only and another containing sterile water with 30 axenic first instars. Both controls were fed alongside experimental treatments to monitor contamination.

Five timepoints were selected to assess both short- and long-term effects of cryopreservation: (i) 24 h, (ii) 10 days, (iii) 16 days, (iv) 30 days, and (v) 6 months. At each timepoint, a cryovial of donor pool (240 μl) was thawed and resuspended in 120 ml of sterile water. For timepoints following the final shipment of donor pools (16 days, 30 days, and 6 months), each institution thawed one vial from its own donor pool (“matched” treatment) and one vial from the other institution’s donor pool (“mismatched” treatment). Flasks were prepared in parallel using axenic larvae sterilized in the same batch (Fig. 1).

**Figure 1:**
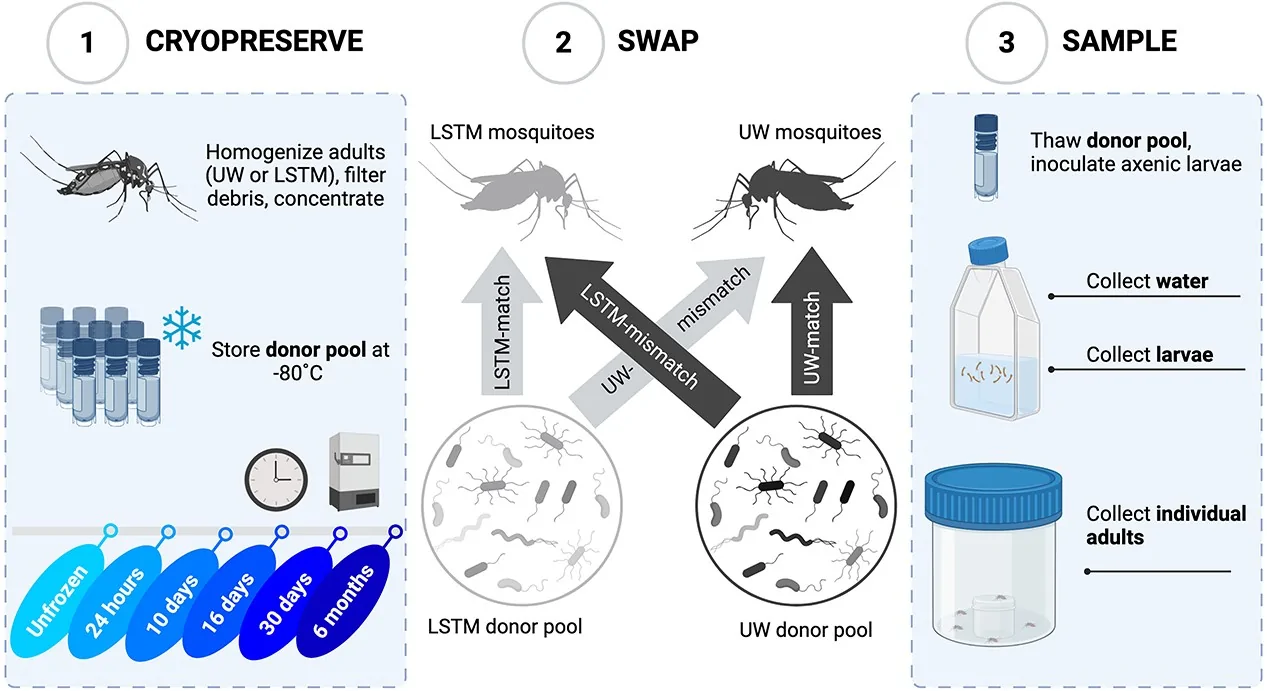
At each institution (UW or LSTM), 40 pools of 18 sugar-fed, 3–4 day old adult female *Ae. aegypti* strain Liverpool mosquitoes were homogenized using 5 mm steel beads in a mechanical homogenizer. Then, cuticle debris was run through a 5 µm filter to generate a donor pool, which was aliquoted into 12 single-use tubes, and stored in 20% glycerol at −80°C for 24 h, 10 days, 16 days, 30 days, or 6 months before a single aliquot of the donor pool was thawed for use. A subset of the filtered donor pool was immediately inoculated into axenic larvae at each institution. Frozen donor pools were shipped on dry ice to each collaborating institution, where on a given timepoint, a single tube of donor pool was thawed and inoculated into axenic L1 larvae. Starting at the 16-day timepoint, parallel flasks were reared with both UW and LSTM donor pools at each institution. Larvae were reared under aseptic conditions on an autoclaved TetraMin diet until pupation, when they were moved to sterile emergence chambers and provided sterile 10% sucrose until the final sampling point at 3–5 days post emergence.

Axenic (germ-free) first instars were generated as previously described [10]. Briefly, conventionally reared *Ae. aegypti* LVP eggs were submerged in 70% ethanol for 5 min, followed by a mixture of 2.5% bleach and 0.1% D-256 disinfectant (Vedco Inc., St Joseph, MO, USA) for 3 min, and then 70% ethanol for 5 min. Eggs were rinsed three times in sterile water and hatched under vacuum. Hatched larvae were rinsed in sterile water and transferred to treatment flasks. Larvae were fed 90 μl of autoclaved powdered TetraMin (100 mg/ml in sterile water) on Days 1, 3, 4, 5, and 7. Water from control flasks was regularly plated on Luria-Bertani (LB) agar (Miller's formulation) to confirm absence of culturable contaminants.

A pool of five third-instars and 1 ml of larval water were collected from each flask on the first day third instars were observed. Upon pupation, mosquitoes were transferred to a sterile emergence chamber containing a pot with 10 ml of sterile water and 5 ml of larval water. Negative controls included additional sterile emergence containers with sterile water and no mosquitoes. Sterile 10% sucrose solution was provided on a cotton wick and refreshed every other day. New pupae were added to the emergence chamber for two consecutive days. After 3–5 days, chambers were cold-anesthetized, and all female mosquitoes were frozen at −80°C for sequencing.

### DNA extraction

DNA from mosquito samples was extracted using a modified QIAamp DNA Mini Kit protocol (Qiagen, Hilden, Germany). Pools of five larvae were surface-sterilized for 1 min in 70% ethanol, rinsed three times in sterile PBS, and stored in PBS at −80°C until extraction. Adult mosquitoes were frozen at −80°C immediately after collection. Prior to extraction, adults were thawed and surface-sterilized for 3 min in 70% ethanol, followed by three 5-min washes in sterile PBS. Abdomens were dissected and placed in bead-beating tubes, while the right wing of each adult was retained on double-sided tape for measurement.

Individual abdomens or larval pools were homogenized with one 5-mm stainless steel bead (Qiagen, Hilden, Germany) in 180 μl buffer ATL using either a bead-beater (20 s; UW) or a TissueLyzer (1 min at 26 Hz; LSTM). Proteinase K (80 μl) was added, and samples were incubated for 3 h before resuming the standard QIAamp DNA Mini protocol.

For larval water, initial donor pools, and all control samples, DNA was extracted using the Qiagen DNeasy Blood & Tissue kit “Field and/or Lab-collected Water” protocol, with an additional 30-min incubation at 37°C in 180 μl enzymatic lysis buffer (20 mM Tris-Cl, pH 8.0; 2 mM sodium EDTA; 1.2% Triton X-100; lysozyme at 20 mg/ml). For all sample types, DNA was eluted in 30 μl nuclease-free water.

DNA concentration was measured using a Quantus fluorometer (Promega, Madison, WI, USA) at UW or a Qubit High Sensitivity Kit at LSTM, and purity was assessed with a NanoDrop spectrophotometer. DNA from LSTM was shipped to UW for library preparation.

### Library preparation and sequencing

DNA extracts were amplified for the V4 region of the bacterial 16S rRNA gene using a previously described one-step amplification with barcoded primers [75]. Each reaction contained 2X DreamTaq HS Master Mix (Thermo Fisher Scientific), 5 pmol of each primer, and ~10 ng/μl of DNA template. Amplification conditions were: initial denaturation at 95°C for 3 min; 30 cycles of 95°C for 45 s, 55°C for 60 s, and 72°C for 30 s; followed by a final extension at 72°C for 10 min. Successful amplification was confirmed by agarose gel electrophoresis. Remaining products were purified using the MagJET NGS Cleanup and Size Selection Kit (Thermo Fisher Scientific) and quantified with a Quantus fluorometer.

A total of 696 libraries were randomly assigned to three sequencing runs to minimize run-to-run variability. Libraries were submitted to the UW Biotechnology Center for paired-end sequencing (2 × 250 bp) on an Illumina MiSeq platform.

### Sequence analysis

Initial data processing was performed separately for each sequencing run. Sequences were imported into QIIME2 for quality control, where forward reads were truncated at 250 bp and reverse reads at 220 bp to remove low-quality trailing bases. DADA2 was applied to each run for quality filtering, denoising, chimera removal, and paired-end merging [76, 77]. After processing, runs were merged, and taxonomy was assigned using a Naïve-Bayes classifier trained on GreenGenes2 (version 2022.10) [78, 79]. Multiple sequence alignment and an unrooted phylogenetic tree were generated using MAFFT and FastTree2 via the align-to-tree-mafft-fasttree command [80, 81]. QIIME2 artifacts were imported into R (version 4.1.3) for downstream analysis, and the phylogenetic tree was rooted based on the longest edge [82]. A phyloseq object was created, and reads not classified as domain “Bacteria” were removed.

Samples underwent stepwise decontamination using the Decontam package [83, 84]. First, samples and experimental controls were grouped by extraction institution (UW or LSTM). The prevalence method (cutoff = 0.5) was used to remove amplicon sequence variants (ASVs) likely introduced during the course of the experiment or during DNA extraction, retaining taxa present at ≥10% relative abundance in ≥5 samples. This step retained 6 of 252 contaminants in LSTM samples and 4 of 331 in UW samples, though two ASVs were manually removed as likely contaminants. A second Decontam run combined all samples and PCR controls to identify contaminants introduced during library preparation, using the prevalence method (cutoff = 0.5) and a threshold of 10% abundance in ≥10 samples; this retained 1 of 38 contaminant ASVs. Samples with fewer than 100 sequences after decontamination were excluded.

Rarefaction curves were generated (Additional File 1, Supplementary material), and a sampling depth of 1042 reads was selected for calculating Shannon’s *H* index using phyloseq. All subsequent analyses were performed on the full, unrarefied phyloseq object [83]. Beta diversity metrics, including robust Aitchison principal-component analysis (RPCA) and phyo-RPCA, were computed using the gemelli plugin in QIIME2 [85]. Differential abundance analysis was conducted on centered log-ratio (CLR)-transformed counts using ALDEx2 [86]. For two-group comparisons, ASVs were considered differentially abundant if *P* < .05 for both the *t-*test and effect modules; Kruskal-Wallis tests were used for comparisons among three or more groups. Additional analyses were performed using custom R scripts (available at https://github.com/kcoonlab/cryo-swap-microbiota-LVP).

### Wing length measurements

Wings of adult female offspring were either mounted on glass slides using double-sided tape (UW) or imaged immediately (LSTM). Images were captured with a Leica S9i digital stereo microscope using Leica Acquire software for macOS and analyzed in ImageJ, calibrated with a stage micrometer. Wing length was measured from the alular notch to the intersection of the radius three vein and outer wing margin, excluding the fringe [87]. Wing length serves as a proxy for adult body size and fecundity [88, 89].

## Results

### High-level taxonomic patterns across donor and recipient samples

After quality control, we obtained a total of 18 936 817 reads from donor pools, larvae, larval water, and adults, with a median sequencing depth of 32 099 reads per sample. Across all samples, we identified 4494 ASVs, with a median of 22 ASVs per sample. Due to the low complexity of these communities, rarefaction curves for most samples reached saturation at fewer than 1000 reads (Additional File 1, Supplementary material).

LSTM-derived samples contained 3008 ASVs, whereas UW-derived samples contained 1796 ASVs. The majority of reads (>99.7%) belonged to the classes Gammaproteobacteria, Bacteroidia, Alphaproteobacteria, Actinomycetia, and Bacilli. UW donor pools were dominated by Gammaproteobacteria, while LSTM donor pools exhibited a mixture of Alphaproteobacteria, Bacteroidia, and Gammaproteobacteria. Downstream LSTM and UW water, larval, and adult samples primarily consisted of Gammaproteobacteria and Bacteroidia, with very few Alphaproteobacteria reads (Additional File 2, Supplementary material).

### Donor pool composition dictates the impact of cryostorage on recipient adult communities

Donor pools were generated by homogenizing 40 pools of 18 conventionally reared, sugar-fed female mosquitoes. These pools represent a snapshot of microbial diversity in adult *Ae. aegypti* strain LVP mosquitoes at each institute. LSTM donor pools contained 729 ASVs, whereas UW donor pools contained 99 ASVs. LSTM pools were dominated by the genera *Asaia* and *Elizabethkingia*, while UW pools consisted almost entirely of an unclassified genus within the family Enterobacteriaceae (Fig. 2).

**Figure 2:**
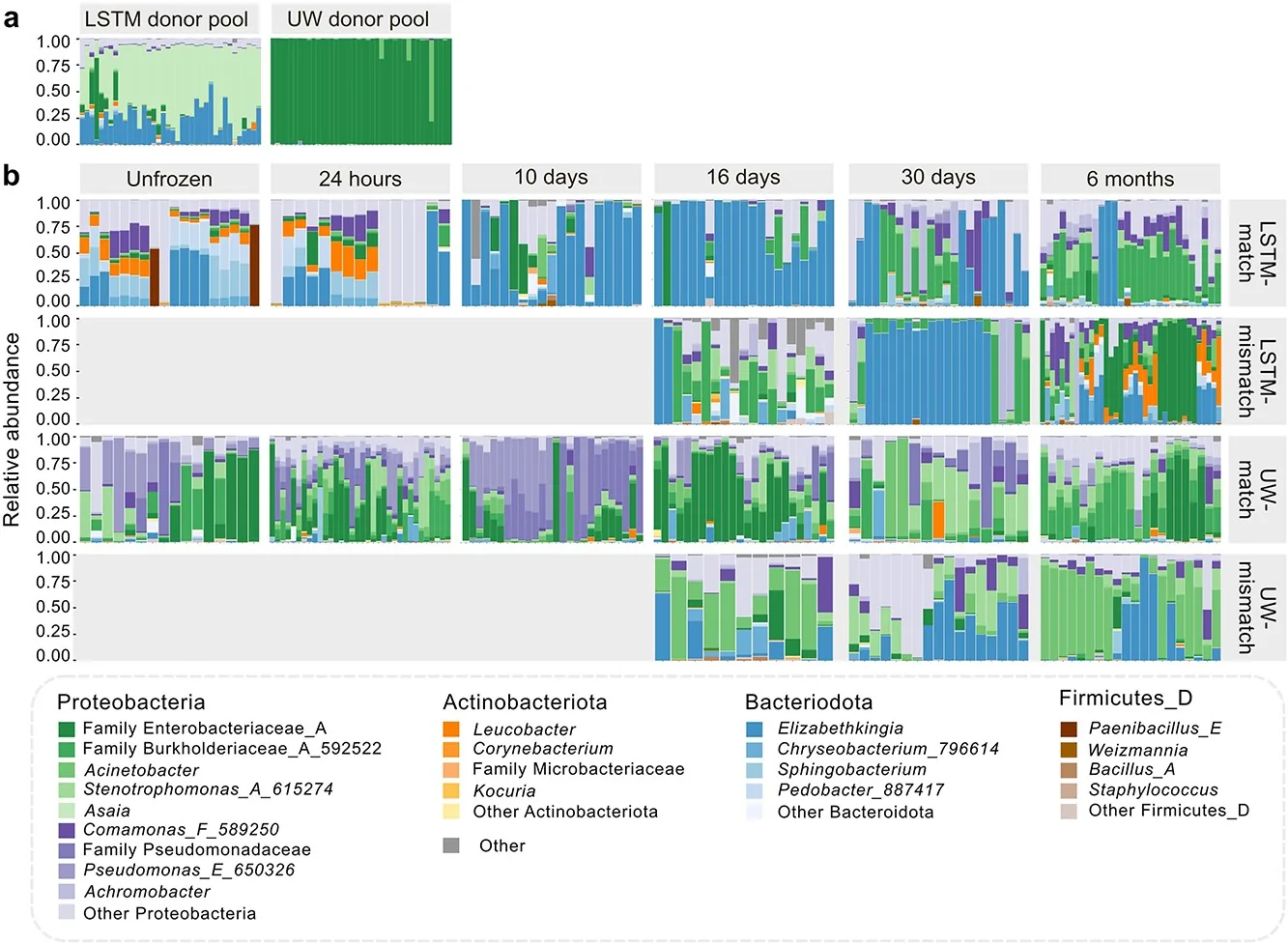
Relative abundance of bacterial taxa in (a) donor pool inputs, where each bar represents 18 female mosquitoes from a laboratory population of *Ae. aegypti* LVP at LSTM or UW, respectively, and (b) recipient adult mosquitoes, where each bar represents an individual adult mosquito. Top labels describe the duration of donor pool storage at −80°C before inoculation into germ-free larval water. The top four genera from the top four bacterial phyla are displayed; additional genera within a phylum, or from other phyla, are classified as “other”.

Donor pools were either immediately inoculated into sterile water containing axenic larvae (the “unfrozen” treatment) or cryopreserved at −80°C in 20% glycerol for 24 h, 10 days, 16 days, 30 days, or 6 months. After storage, a single aliquot was thawed and added to axenic larvae, which developed to adulthood under aseptic conditions (Fig. 1). We define four treatments: UW-match, UW-mismatch, LSTM-match, and LSTM-mismatch. These refer to the colony identity of the recipient mosquitoes (UW or LSTM) and whether the donor pool originated from the same colony (match) or from the colony maintained at the other institution (mismatch).

A primary goal of microbiota transplantation is to ensure that recipients are colonized by a diverse bacterial consortium from the donor. We first assessed alpha diversity using Shannon’s *H* index, which accounts for both richness and evenness. In adult mosquitoes, Shannon’s index varied over time but did not decrease monotonically in either UW or LSTM groups (Additional File 3, Supplementary material). This suggests that longer cryostorage does not necessarily result in less diverse communities. In UW-match adults, none of the timepoints differed significantly from the unfrozen treatment (Dunn’s test, *Z* < 2.22, *P* > .05). In LSTM-match adults, diversity was lower at 10, 16, and 30 days (*Z* > 3.27, *P* < .01), but after 6 months, diversity was comparable to the unfrozen treatment (*Z* = 1.29, *P* = 1.00). UW-mismatch adults showed no significant variation (Kruskal-Wallis, *H*_2_ = 4.26, *P* = .12), whereas LSTM-mismatch adults were less diverse at Day 30 (Dunn’s test, *Z* > 4.27, *P* < .0001), likely due to an *Elizabethkingia* bloom (Fig. 2).

To evaluate whether cryostorage duration influenced similarity between donor pools and recipient adults, we examined beta diversity (Additional File 4, Supplementary material). Both UW-match and LSTM-match adults were more dissimilar from their donor pools after 6 months compared to the unfrozen treatment [Tukey’s honest significant difference (HSD), *P* < .0001]. However, the unfrozen treatment was never the most similar to the donor pool, indicating that cryostorage time alone does not determine community similarity (Additional File 4, Supplementary material).

Consistent with previous transplant studies [67, 69, 90], recovery of individual taxa was uneven. *Asaia*, dominant in LSTM donor pools, was virtually absent in adult samples. *Asaia* was undetected in 362 of 389 adults and never exceeded 165 reads (0.5%). In contrast, *Elizabethkingia* persisted across all LSTM timepoints, though its abundance varied among replicates and individuals. Differential abundance analysis revealed that in LSTM-match adults, *Vibrio* and *Leucobacter* spp. were more common at early timepoints, while *Stenotrophomonas, Agrobacterium, Achromobacter*, and members of *Burkholderiaceae* were enriched later (ALDEx2, Kruskal-Wallis *P* < .05). Notably, these taxa were rare in donor pools. UW-match adults did not exhibit taxa correlated with cryostorage duration; despite the simple UW donor community, UW-match adults harbored diverse taxa that were present but rare in the donor pool (Fig. 2).

### Recipient adults respond differently to transplants from matched vs. mismatched donor mosquitoes

We compared alpha diversity (Shannon’s *H* index) across the four combinations of university and match/mismatch treatments. Microbial communities in UW-match adults exhibited significantly higher diversity than both LSTM-match and LSTM-mismatch treatments (Kruskal-Wallis, *H*_3_ = 19.264, *P* = .0003; Dunn’s test, *P* < .05). UW-mismatch samples showed intermediate diversity that was not significantly different from other treatments (Fig. 3b).

**Figure 3:**
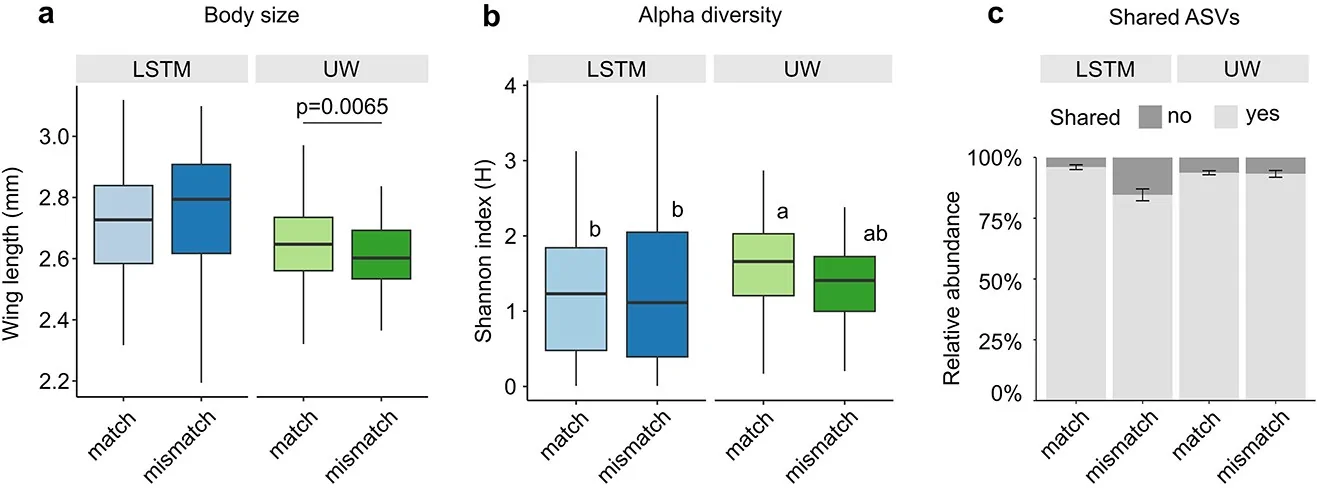
(a) Significant interaction between institution (LSTM vs. UW) and treatment (match vs. mismatch) on wing length (linear model, *P* = .006); UW-mismatch wings are significantly smaller than UW-match, while LSTM groups did not differ. Panel (b) shows differences in alpha diversity as measured by Shannon’s *H* index. Treatments that share a letter do not have differences in alpha diversity (Kruskal-Wallis test and Dunn’s test, *P <* .05). (c) Relative abundance of reads in adult mosquitoes by treatment, each read colored by whether a given ASV was detected in the pertinent donor pool. LSTM-match adults had the highest percentage of reads shared with the donor pool, and UW-match adults also shared more than UW-mismatch (Kruskal-Wallis test; *H*_3_ = 49.09, *P* < .001; Dunn’s test; *P* < .05). LSTM-mismatch samples had the most variability in this measure (Levene’s test, *P* < .001). Error bars represent standard error of the mean.

Given the large diversity difference between LSTM and UW donor pools, we next assessed whether adult samples were composed of ASVs detected in the corresponding donor pool. Across treatments, >92% of reads (median = 98%) originated from ASVs present in the donor pool (Fig. 3c). LSTM-match adults had the highest percentage of shared reads, and UW-match adults also shared more than UW-mismatch (Kruskal-Wallis test, *H*_3_ = 49.09, *P* < .001; Dunn’s test, *P* < .05). This suggests that matched treatments retain more common donor taxa, whereas mismatched treatments include more ASVs that are rare or undetected in donor pools. LSTM-mismatch samples displayed the greatest variance in shared ASV abundance (Levene’s test, *P* < .0001).

Finally, we examined whether wing length was influenced by microbial transplant treatments. Wing length was significantly affected by an interaction between host strain (UW vs. LSTM) and match/mismatch treatment (linear model, *β* = −0.104, *P* = .0006). UW-mismatch mosquitoes had smaller wings than UW-match mosquitoes (Fig. 3a, *P* = .006), whereas LSTM-mismatch resulted in a slight, non-significant increase (*P* = .0734). We also tested for correlations between specific ASVs and wing size but found none (aldex.corr, *P* > .05).

We analyzed beta diversity using RPCA, which accounts for the compositional nature of 16S rRNA data (Fig. 4) [85]. All groups were significantly different (Additional File 5, Supplementary material; Permutational Multivariate Analysis of Variance [PERMANOVA], *F*_5,383_ = 57.56, *P* < .001), except LSTM-match and LSTM-mismatch adults, which clustered together (*F*_1,197_ = 2.94, *P* = .615). This striking result suggests that UW adult communities are strongly influenced by donor source, whereas LSTM adults exhibit more variable communities less affected by donor origin. Phylo-RPCA, which incorporates phylogenetic relatedness, produced similar results (PERMANOVA, *F*_5,383_ = 72.47, *P* < .001). Notably, group dispersion was unequal, with LSTM samples showing significantly greater spread (Additional File 5, Supplementary material).

**Figure 4:**
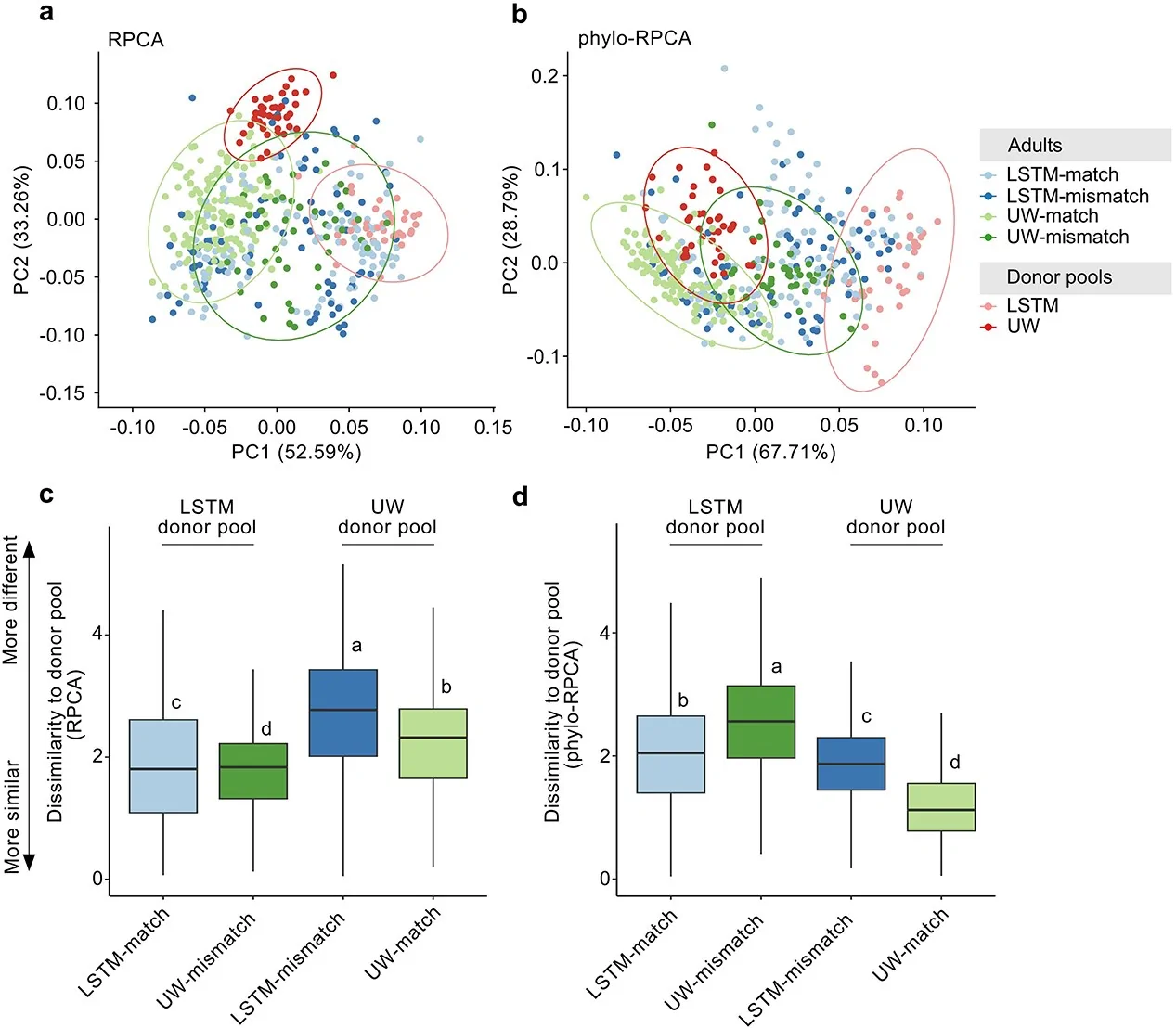
Differences in beta diversity of adult mosquitoes in relation to donor pools displayed by (a) RPCA and (b) phylo-RPCA. All groups significantly different (PERMANOVA, *P* < .05) with the exception of LSTM-match versus LSTM-mismatch adults (Additional File 4, Supplementary material). Ellipses generated assuming a multivariate normal distribution. Comparison of RPCA (c) and phylo-RPCA (d) distance between individual adult mosquitoes and their corresponding donor pool, with a greater score indicating higher dissimilarity from the donor. Treatments that share a letter do not have differences in alpha diversity as measured by Analysis of Variance (ANOVA) with Tukey’s HSD (*P* < .001).

All adult groups clustered separately from both donor pools (*P* < .05). UW mosquitoes were more similar to the UW donor pool than the LSTM mosquitoes receiving the same donor pool, based on both RPCA and phylo-RPCA metrics (Tukey’s HSD, *P* < .0001). However, whether UW or LSTM recipients resembled the donor pool more closely depended on whether the metric accounted for phylogeny (Fig. 4c and d).

To assess the effects of swap treatment, cryostorage duration, and stochastic assembly among replicate flasks, we performed a PERMANOVA on RPCA distances. Treatment, timepoint, and their interaction were all significant (*P* < .001, Additional File 5, Supplementary material). Variation among replicate flasks within each treatment-timepoint combination explained a substantial fraction of variation (*F*_53,313_ = 3.57, *R*^2^ = 0.22, *P* < .001). Dispersion differed significantly among treatments and flasks but not among timepoints (Permutational Multivariate Analysis of Dispersion [PERMDISP]; Additional File 5, Supplementary material). Similar trends were observed for phylo-RPCA distances.

### Engraftment of specific donor bacteria varies between recipient mosquito strains

Given the clear differences in microbial communities by mosquito strain and match/mismatch treatment, we used ALDEx2 for differential abundance analysis to identify taxa associated with these trends. Regardless of donor source (UW or LSTM), adult UW mosquitoes were consistently enriched in *Acinetobacter, Pseudomonas, Stenotrophomonas, Comamonas*, and *Achromobacter* (Fig. 5). Interestingly, while the UW-match and UW-mismatch adults shared these genera, they differed at the ASV level, suggesting that UW mosquitoes consistently associate with certain genera despite species- or strain-level variation in donor pools.

**Figure 5:**
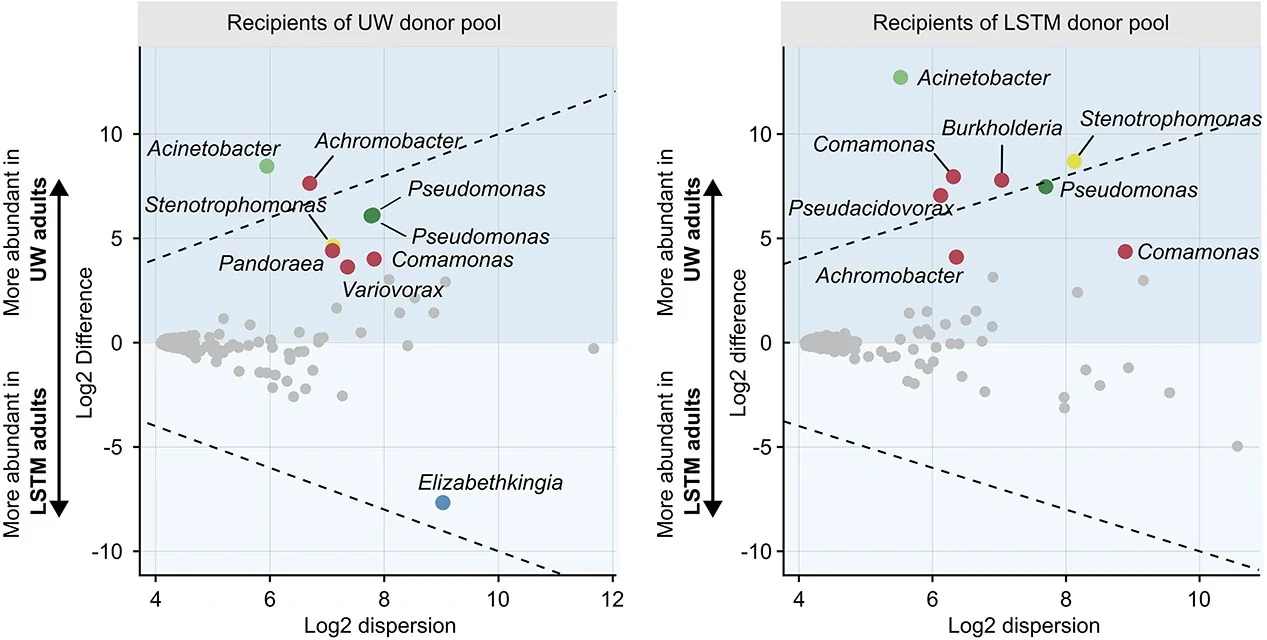
Differentially abundant ASVs in UW and LSTM adult mosquitoes that received UW (a) or LSTM (b) donor pools. Significant taxa are represented with a genus label and a dot colored by the family-level classification (ALDEx2, Welch’s *t-*test, *P* < .05). Note significant ASVs do not overlap in panels a and b, but the same genera are shared across panels.

Differential abundance analysis identified *Elizabethkingia* as significantly enriched in LSTM adults compared to UW adults among mosquitoes that received UW donor pools (Fig. 5a); this enrichment was not detected among mosquitoes that received LSTM donor pools, since *Elizabethkingia* had already colonized both LSTM-match and UW-mismatch adults in that comparison (Fig. 5b, Fig. 2). Notably, *Elizabethkingia* was nearly absent from UW-match water and larval samples and detected only at very low abundance in UW-match adults (Additional File 6, Supplementary material).

To explore factors shaping variation in *Elizabethkingia* abundance, we performed Spearman’s rank correlations among the 25 most abundant ASVs in LSTM-match, LSTM-mismatch, UW-mismatch, and UW-match adults (Additional Files 7 and 8, Supplementary material). *Elizabethkingia* abundance was positively correlated with *Comamonas_d325* in LSTM adults (*ρ* > 0.52, *P* < .0001) but not in UW-mismatch adults (*ρ* = 0.084, *P* = 1.00). These ASVs also exhibited similar interaction patterns with other taxa (Additional File 7, Supplementary material). In contrast, this correlation was absent in water samples and only observed in UW-mismatch larvae (*ρ* = 0.81, *P* = .045; Additional File 9, Supplementary material). A separate *Comamonas* ASV (*Comamonas_16da*) was negatively correlated with *Elizabethkingia* in LSTM adults (*ρ* < −0.68, *P* < .0001) but showed no significant interaction in UW-mismatch adults (*ρ* = −0.134, *P* = 1.00; Additional Files 10 and 11, Supplementary material). Additional negative correlations in LSTM adults included *Stenotrophomonas* (*ρ* < −0.54), *Achromobacter* (*ρ* < −0.53), and *Agrobacterium* (*ρ* < −0.62). In UW-mismatch adults, correlations with *Elizabethkingia* were weaker and fewer, including slight negative associations with *Acinetobacter* (*ρ* = −0.37), *Burkholderia* (*ρ* = −0.33), and *Pseudomonas* (*ρ* = −0.39).

Adult microbial communities were further grouped into two biotypes based on PAM clustering on RPCA distances at the ASV level (Additional File 12, Supplementary material). *Elizabethkingia* abundance was the primary differentiator between these biotypes. Differential abundance analysis revealed that Biotype 1 (not dominated by *Elizabethkingia*) was enriched in *Pseudomonas, Comamonas, Acinetobacter*, and ASVs classified only to family level within *Pseudomonadaceae* and *Burkholderiaceae*.

Finally, we examined taxa that were less successful at colonizing mismatched hosts. We defined “successfully transplanted taxa” as those present in donor pools and detected in at least half of recipient flasks. For LSTM donor pools, 36 taxa met this criterion in LSTM-match flasks; six of these were absent from all UW-mismatch samples (Additional File 13, Supplementary material). The most abundant of these was *Agrobacterium*, though most were rare in LSTM-match adults (Additional File 14, Supplementary material). For UW donor pools, 24 taxa were detected in at least half of UW-match flasks, and only one (*Arthrobacter*) was absent from LSTM-mismatch flasks; this ASV never exceeded 4% of reads in UW-match adults (Additional File 15, Supplementary material).

### Mosquito life stage influences microbial community composition

Mosquitoes are holometabolous insects with an aquatic larval stage, which can interrupt bacterial transmission from larvae to adults. This experimental design uniquely examines community assembly across the mosquito life cycle in a closed system, where bacteria must persist in water or larvae to successfully colonize adults.

To assess shifts in microbial composition, we analyzed beta diversity. Water and larval samples clustered together across all treatments in both RPCA and phylo-RPCA metrics, except for UW-mismatch RPCA (Additional Files 16-18, Supplementary material). Adult samples generally clustered separately from water and larvae, with one exception: LSTM-match adults clustered with larvae by RPCA and with both water and larvae by phylo-RPCA (Additional File 18, Supplementary material).

Differential abundance analysis identified taxa contributing to these stage-specific patterns. In UW-match samples, two ASVs (unclassified *Pseudomonadaceae* and *Burkholderiaceae*) were more abundant in adults compared to aquatic samples (larval water and larvae, Additional File 19, Supplementary material). UW-mismatch adults were enriched in *Stenotrophomonas.* In both UW-match and UW-mismatch treatments, aquatic samples contained more of the unclassified *Enterobacteriaceae* ASV and *Leucobacter* (Additional Files 19 and 20, Supplementary material). Neither LSTM-match nor LSTM-mismatch treatments showed differentially abundant ASVs between aquatic and adult stages, likely due to high inter-individual variation in both larvae and adults.

UW water and larval samples were highly similar (Additional File 21, Supplementary material). Across all frozen timepoints, larval water was dominated by the same unclassified *Enterobacteriaceae* ASV found in the donor pool (Fig. 2, Additional File 21, Supplementary material), with smaller proportions of *Leucobacter, Chryseobacterium,* and *Pedobacter* remaining consistent over time. Larval samples were similar but contained fewer Bacteroidota reads. In UW-match samples, *Chryseobacterium* was more abundant in larval water, while *Leucobacter* was more abundant in larvae (ALDEx2_ttest_ and ALDEx2_effect_, *P* < .05; Additional File 22, Supplementary material). Unfrozen UW donor pools produced larvae dominated by *Leucobacter* (~75% of reads), while water contained higher proportions of *Pedobacter, Chryseobacterium*, and *Sphingobacterium* (Additional File 21, Supplementary material). UW-mismatch water and larvae resembled UW-match samples but included substantial *Elizabethkingia* from the LSTM donor pool. UW-mismatch larval water contained more *Pedobacter* than larvae (ALDEx2_ttest_ and ALDEx2_effect_, *P* < .05; Additional File 23, Supplementary material).

LSTM water and larvae from unfrozen donor pools consistently contained *Elizabethkingia, Pedobacter, Leucobacter*, and an unclassified *Enterobacteriaceae* (Additional File 24, Supplementary material). Frozen timepoints exhibited more stochastic community assembly, mirroring patterns observed in adult LSTM mosquitoes. *Elizabethkingia* often dominated, accounting for >75% of reads in individual water or larval samples. No ASVs were differentially abundant between larval water and larvae in either LSTM-match or LSTM-mismatch treatments (ALDEx2_ttest_ and ALDEx2_effect_, *P* > .05).

## Discussion

Using two long-established laboratory colonies of *Ae. aegypti*—each maintained in isolation for almost 50 years—we demonstrate that cryopreserved mosquito microbiota transplants can reveal host-specific responses to microbial communities. Our reciprocal transplant revealed an unexpected trend: alpha and beta diversity in UW adults were significantly influenced by whether they received mismatched microbiota, whereas LSTM adult diversity was not primarily driven by donor source. To identify taxa underlying these host-specific effects, we examined ASVs associated with different host backgrounds receiving the same donor pool. UW mosquitoes were consistently associated with *Acinetobacter, Achromobacter, Pseudomonas, Comamonas*, and *Stenotrophomonas* regardless of donor origin. Interestingly, while these genera were shared across UW-match and UW-mismatch adults, they represented distinct ASVs, suggesting genus-level competency at persisting in the UW host environment. Conversely, LSTM-match adults were associated with *Elizabethkingia*, which was absent from UW-match treatments. These microbial differences corresponded to a phenotypic effect: UW mosquitoes receiving mismatched donor pools had smaller wings, a reliable proxy for adult body size and overall quality [88, 89]. No individual taxa in water or larval samples correlated with this developmental phenotype.

Although the microbiota is known to influence mosquito survival and fecundity [11–15], we did not directly measure these traits in the present study. Adults were sampled destructively for microbiome and wing-length analysis, precluding downstream assessment of survival or reproductive output (e.g. egg production and hatch rate) in the same individuals. Pairing microbiota transplantation with non-destructive or longitudinal measures of host fitness represents an important direction for future work, allowing the strain- and donor-specific patterns of microbial assembly reported here to be linked directly to fitness outcomes.

Differences in how UW and LSTM mosquitoes respond to microbiota transplants likely reflect host genetic divergence after decades of laboratory maintenance. Previous work shows that *Ae. aegypti* colonies derived from wild populations converge on similar microbial communities under laboratory conditions, regardless of insectary location, highlighting the strong influence of the environment. However, within a single insectary, genetically distinct lines harbor distinct microbial profiles [91, 92]. Recent studies confirm that while the insectary environment is the dominant driver of microbial composition, host signatures remain detectable [71]. Together, these findings support a model of host-by-environment interactions shaping mosquito microbiota. Our study minimized environmental contributions by transplanting adult-derived communities into axenic larvae and rearing them under controlled conditions, revealing that two genetically distinct hosts maintain unique relationships with their microbiota—mediated by host genetics and stochastic assembly processes.

We also note that reciprocal transplants were performed at each mosquito line’s home institution (LSTM donor pools into UW recipients at UW, and vice versa) rather than at a single shared site. This design does not fully disentangle host genetic background from potential laboratory-specific environmental effects on the transplanted communities. Axenic larvae were reared under standardized, aseptic conditions using identical diet and protocols at both sites, which should limit this confound relative to the substantial rearing differences accumulated between colonies over five decades; nonetheless, future studies performing reciprocal transplants at a single shared facility would help fully isolate host-genotype effects from residual site-specific influences.

In addition to host–microbe interactions, we examined microbe−microbe interactions [91, 93, 94]. Several bacterial taxa negatively correlated with *Elizabethkingia* abundance in adult mosquitoes, including *Stenotrophomonas, Achromobacter*, and *Agrobacterium* in LSTM mosquitoes, and *Acinetobacter, Burkholderia*, and *Pseudomonas* in UW mosquitoes. Overall, Spearman correlation strengths were weaker in UW adults, suggesting that community assembly in LSTM adults may be more strongly influenced by bacterial interactions, whereas assembly in UW adults may depend more on host factors such as immune responses.

*Elizabethkingia* is commonly associated with mosquitoes and is considered a promising candidate for vector control [95, 96]. It exhibits anti-*Plasmodium* properties [97] and proliferates following blood feeding [96, 98, 99], making it important to understand how bacterial competition and host factors influence its abundance and persistence, factors critical for field deployment. These interactions will also affect the efficacy of other microbial inoculants. In our study, *Elizabethkingia* dominated many LSTM adult samples, where it showed both negative and positive correlations with different *Comamonas* ASVs. Independent biotype analysis revealed two major clusters, one strongly enriched in *Elizabethkingia*, suggesting that this taxon may shape overall community composition.

Interestingly, *Elizabethkingia* was detected at extremely low abundance in UW donor pools but was prevalent in LSTM-mismatch mosquitoes and nearly absent from UW-match mosquitoes that received the same donor pools. Its presence in UW-mismatch mosquitoes indicates that exclusion from UW-match mosquitoes is not solely due to host-derived factors; rather, its abundance likely depends on interactions with other microbes during larval or adult stages.

An alternative, non-mutually-exclusive explanation for the *Elizabethkingia* patterns described above is that divergent insectary conditions at UW and LSTM, rather than microbial community interactions alone, shape engraftment success. Consistent with this possibility, a related study from our group found that *Ae. aegypti* reared in different insectaries within the same institution harbor significantly divergent microbiomes, with insectary identity being a stronger predictor of composition than host genotype [72]. In our dataset, *Elizabethkingia* was highly abundant in LSTM-reared adults regardless of donor source, suggesting a strong insectary and/or host-genetic contribution to its persistence at LSTM. At UW, however, *Elizabethkingia* became dominant only in mosquitoes that received an LSTM-derived donor pool, indicating that UW insectary conditions can support this taxon, but apparently only in the presence of an LSTM-associated microbial community. We therefore favor an interpretation in which insectary/host effects and microbial-community effects jointly shape *Elizabethkingia* engraftment, rather than either factor acting alone. Disentangling these contributions further—e.g. by performing reciprocal transplants within a single insectary or standardizing rearing conditions across sites—represents an important direction for future work building on the reciprocal-transplant design introduced here.

Our experiment involved a large-scale reciprocal transplantation conducted between two universities over 6 months. A key objective was to test whether storing adult-derived donor pools at −80°C for extended periods (up to 6 months) would affect the recovery of taxa in recipient mosquitoes. We observed differences in alpha and beta diversity among adults reared from donor pools cryopreserved for different durations; however, these differences did not consistently trend away from the donor pool or toward reduced alpha diversity. This interpretation is supported by significant replicate-level effects, which explained a substantial proportion of variation even after accounting for treatment, cryostorage duration, and their interaction. Our findings are consistent with observations from mammalian transplant systems. In fecal microbiota transplantation, freezing donor stool alters overall community diversity and affects the engraftment of several bacterial groups, including Actinobacteria, Bacteroidetes, and Lachnospiraceae [90, 100, 101]. Donor microbiota viability in these systems is influenced by factors such as cryoprotectant use, duration of storage at −80°C, and the method used for thawing [102–104]. Despite these effects, clinical trials and a recent meta-analysis demonstrate that frozen donor material retains high efficacy for treating recurrent *Clostridium difficile* infection, indicating that frozen microbiota continue to perform key community functions [105–107].

Recipient adult bacterial communities were never identical to donor pools, even when unfrozen donor pools were transplanted into axenic larvae from the same mosquito population. This outcome is unsurprising given the holometabolous life cycle of mosquitoes, which includes an aquatic larval stage where microbes experience selective pressures distinct from those shaping adult communities. Each replicate cohort encountered multiple transitions and bottlenecks capable of driving divergence, including: (i) initial bacterial bloom and competition after inoculation, (ii) interactions with the larval gut and aquatic environment, (iii) host tissue remodeling during pupation, (iv) gut re-inoculation when adults imbibe larval water, and (v) transmission via contact among co-housed adults. Each stage represents an opportunity for microbial communities to follow different trajectories, even when sourced from the same donor pool.

One striking example of loss during transplantation was *Asaia*, which comprised 58% of reads in the LSTM donor pool yet was nearly absent from all downstream samples—even when donor pools were not cryopreserved. This likely reflects selective pressures during the larval stage as *Asaia* is a vertically transmitted microbe that colonizes adult guts and reproductive tissues and readily persists in nectar sources but poorly colonizes aquatic environments [108–112]. *Leucobacter* showed a different pattern: although less common in later timepoints of LSTM-match adults, it persisted in LSTM-mismatch adults after 6 months of cryostorage and was detected in all UW larval samples. This suggests that *Leucobacter* can survive cryostorage and aquatic environments but is outcompeted in certain adult assemblages. *Leucobacter* is frequently detected in larvae and larval habitats of both wild and laboratory-reared *Aedes* mosquitoes [10, 15, 113–119].

Laboratory mosquito colonies are essential for advancing our mechanistic understanding of mosquito biology and pathogen transmission. However, their microbial communities differ substantially from those in wild mosquitoes. Given growing evidence that microbiota influence mosquito survival, fecundity, insecticide resistance, and vector competence, strategies that better approximate naturally acquired microbial communities in the laboratory are needed to bridge the gap between laboratory and field conditions.

It is important to note that the transplantation approach described here captures only one dimension of the ecological processes that shape microbial communities in nature. Wild mosquito microbiota assembly is also driven by factors absent from our closed laboratory system, including natural nutritional resources (e.g. diverse floral and larval detritus sources), continuous exposure to environmental microbial reservoirs, and fluctuating abiotic conditions. Controlled microbiota transplants are therefore best viewed as a complementary tool for isolating host-genotype and donor-community effects, rather than a stand-alone strategy for “rewilding” laboratory mosquito microbiomes. Realizing that broader goal will likely require integrating microbiota transplantation with ecologically relevant dietary and environmental manipulations that better approximate how natural microbial communities are assembled and maintained in wild mosquito populations.

The transplantation method described here enables reciprocal microbiota exchange between laboratory colonies across time and space and offers a foundation for introducing field-derived microbiota into laboratory mosquitoes. Developing this approach will improve confidence that phenotypes observed in laboratory settings translate to wild populations. Future studies to replicate donor communities more precisely might consider inoculating adults directly via sucrose wicks [9, 120] bypassing selective pressures during the aquatic stage. However, differential bacterial survival and replication in sucrose meals should also be evaluated.

## Conclusion

This study introduces a novel method for collecting, cryopreserving, and transplanting microbial communities from adult mosquitoes into axenic larvae. We validated that this approach establishes diverse microbial communities in recipient adults and revealed host-dependent patterns of community assembly. Cryopreserved adult mosquito microbiota position this system as a tractable model for investigating host–microbe interactions in a transient context. Beyond its experimental utility, this method offers a standardized framework for microbiota transplantation across laboratories and, ultimately, a reproducible strategy for introducing field-derived microbiota into laboratory mosquito populations.

## Supplementary Material

Supplementary_materials_aazag010

## Data Availability

Raw Illumina reads are available in the NCBI Sequence Read Archive (http://www.ncbi.nlm.nih.gov/sra) under BioProject ID PRJNA1432904. Scripts used for analysis and figure generation are available in the following GitHub repository: https://github.com/kcoonlab/cryo-swap-microbiota-LVP. All other data generated by this study are available as Supporting Information herein.
