# Supplementary material for: Transplant of cryopreserved mosquito gut microbiota reveals strain-specific patterns of microbial acquisition and assembly
Source: ISME Host Microbe. 2026 Sep 18;1(1):aazag010. doi: 10.1093/ismehm/aazag010 (PMC13637714; doi:10.1093/ismehm/aazag010)
Supplement: Supplementary file 1 [file supplementary_materials_aazag010.zip › Supplementary_Figures_ISMEHM-D-26-00028.docx]

**Additional Files**

*Transplant of cryopreserved mosquito gut microbiota reveals strain-specific patterns of microbial acquisition and assembly*

Nichols, Dhokiya, Hoque, Heinz, Hughes, Coon

**Additional File 1:** (a) Rarefaction curves demonstrating the saturation of species richness (number of ASVs) detected at the chosen rarefaction depth of 1042 reads. Rarefied data were used only for calculating Shannon's H Index (Figure 4, Additional File 2). (b) Rarefaction curves to a depth of the median number of reads per sample (32,099) demonstrates that sequencing effort was sufficient to saturate rarefaction curves for all sample types.

**Additional File 2:** Stacked bar plot of relative abundance of reads, classified at Class level. Each donor pool sample is composed of 18 pooled 3-4 day post emergence female mosquitoes, larval water is 1 mL larval water from water containing L3 larvae, larval samples are pools of 5 L3-L4 larvae, and adults samples represent individual sugar-fed female adults at 3-5 days post emergence.

**Additional File 3:** Alpha diversity (Shannon's H Index) of adult mosquitoes by duration of donor pool cryostorage. Unfrozen treatment represents immediate inoculation of larval flasks after donor pool was generated, while other time points represent the duration that a portion of the donor pool was stored at -80C before it was thawed and inoculated into larval flasks. Alpha diversity of adult mosquitoes is analyzed separately for each combination of institution (LSTM or UW) and treatment (matched or mismatched). Treatments that share a letter do not have differences in alpha diversity as measured by Kruskal-Wallis test followed by Dunn's test with Bonferroni's correction. (p < 0.05, n.s. = not significant).

**Additional File 4:** Comparison of RPCA (a,b) and phylo-RPCA (c,d) distance between individual adult mosquitoes and their corresponding donor pool, with a greater score indicating higher dissimilarity from the donor. Treatments that share a letter do not have differences in alpha diversity as measured by ANOVA with Tukey's Honest Significant Difference (p < 0.001).

**Additional File 6:** Relative abundance of Elizabethkingia ASV reads in (a) all recipient samples and (b) donor pool samples. Different colors represent different Elizabethkingia ASVs.

**Additional File 7:** Spearman's rank correlation between the 25 most abundant ASVs by read count in (a) LSTM-match (b) and LSTM-mismatch adult samples. Comamonas ASVs have been highlighted with asterisk, and the first 5 characters of the ASV label have been added to distinguish ASVs with the same genus name.

**Additional File 8:** Spearman's rank correlation between the 25 most abundant ASVs by read count in (a) UW-match (b) and UW-mismatch adult samples. Comamonas ASVs have been highlighted with asterisk, and the first 5 characters of the ASV label have been added to distinguish ASVs with the same genus name.

**Additional File 11:** Relative abundance of Elizabethkingia, Comamonas_d32e5, and Comamonas_16da9 ASV reads in adult samples.

**Additional File 12:** (a) Relative abundance of bacterial reads in the two biotypes from PAM clustering analysis on individual adult samples. (b) Beta diversity (RPCA) of individual adult samples. Colors represent treatment, and shapes represent biotype.

**Additional File 14:** Relative abundance of “Lost from LSTM donor pool” taxa in adult samples. Colored taxa represent ASVs that were in the LSTM donor pool and were detected in adults from at least half of LSTM-match flasks, but were not detected in any UW-mismatch adult samples.

**Additional File 15:** Relative abundance of “Lost from UW donor pool” taxa in adult samples. Colored taxa represent ASVs that were in the UW donor pool and were detected in at least half of UW-match flaks, but were not detected in any LSTM-mismatch adult samples. Note that the x-axis tops out at 0.5% of reads in a given sample.

**Additional File 16:** Differences in beta diversity of larval water (dark blue), larvae (light green), and adult (light blue) samples displayed by robust principal component analysis (RPCA). Water and larvae samples clustered together in all treatments (PERMANOVA, p > 0.05) except for UW-mismatch (p = 0.009). Adults clustered separately from both water and larvae (p < 0.05), except for LSTM-match adults, which were not significantly different from water (p = 0.072). Ellipses generated assuming a multivariate normal distribution.

**Additional File 17:** Differences in beta diversity of larval water (dark blue), larvae (light green), and adult (light blue) samples displayed by phylogenetically-aware robust principal component analysis (phylo-RPCA). Water and larvae samples clustered together in all treatments (PERMANOVA, p > 0.05). Adults clustered separately from both water and larvae (PERMANOVA, p < 0.05), except for LSTM-match adults, which were not significantly different from larvae or water (PERMANOVA, p > 0.1). Ellipses generated assuming a multivariate normal distribution.

**Additional File 19:** Relative abundance plots comparing UW-match adults vs aquatic (Water + larvae) with ALDEx2, including only taxa determined to be significantly associated with stage via both ttest and effect modules (p< 0.05).

**Additional File 20:** Relative abundance plots comparing UW-mismatch adults vs aquatic (Water + larvae) with ALDEx2, including only taxa determined to be significantly associated with stage via both ttest and effect modules (p< 0.05).

**Additional File 21:** Relative abundance stacked bar plots comparing UW larvae and water samples by duration of donor pool cryostorage. Unfrozen treatment represents immediate inoculation of larval flasks after donor pool was generated, while other time points represent the duration that a portion of the donor pool was stored at -80˚C before it was thawed and inoculated into larval flasks. UW-match treatments have gray headers, UW-mismatch treatments have black headers. Blank bars are due to errors in sample handling.

**Additional File 22:** Relative abundance plots comparing UW-match water vs larvae with ADLEx2, including only taxa determined to be significantly associated with water or larvae sample via both ttest and effect modules (p< 0.05)

**Additional File 23:** Relative abundance plots comparing UW-mismatch water vs larvae with ALDEx2, including only taxa determined to be significantly associated with water or larvae sample via both ttest and effect modules (p< 0.05)

**Additional File 24:** Relative abundance stacked bar plots comparing LSTM larvae and water samples by duration of donor pool cryostorage. Unfrozen treatment represents immediate inoculation of larval flasks after donor pool was generated, while other time points represent the duration that a portion of the donor pool was stored at -80˚C before it was thawed and inoculated into larval flasks. LSTM-match treatments have gray headers, LSTM-mismatch treatments have black headers.
